# Outlier identification and monitoring of institutional or clinician performance: an overview of statistical methods and application to national audit data

**DOI:** 10.1186/s12913-022-08995-z

**Published:** 2023-01-10

**Authors:** Menelaos Pavlou, Gareth Ambler, Rumana Z. Omar, Andrew T. Goodwin, Uday Trivedi, Peter Ludman, Mark de Belder

**Affiliations:** 1 Department of Statistical Science, UCL, London, UK; 2 Department of Cardiothoracic Surgery, South Tees Hospitals NHS Foundation Trust, Middlesbrough, UK; 3 National Institute for Cardiovascular Outcomes Research (NICOR), Barts Health NHS Trust, London, UK; 4 Department of Cardiac Surgery, University Hospital Sussex NHS Foundation Trust, Brighton, UK; 5 Institute of Cardiovascular Sciences, University of Birmingham, Birmingham, UK

**Keywords:** Outlier detection, Funnel plot, Random effects model, Overdispersion

## Abstract

**Background:**

Institutions or clinicians (units) are often compared according to a performance indicator such as in-hospital mortality. Several approaches have been proposed for the detection of outlying units, whose performance deviates from the overall performance.

**Methods:**

We provide an overview of three approaches commonly used to monitor institutional performances for outlier detection. These are the common-mean model, the ‘Normal-Poisson’ random effects model and the ‘Logistic’ random effects model. For the latter we also propose a visualisation technique. The common-mean model assumes that the underlying true performance of all units is equal and that any observed variation between units is due to chance. Even after applying case-mix adjustment, this assumption is often violated due to overdispersion and a post-hoc correction may need to be applied. The random effects models relax this assumption and explicitly allow the true performance to differ between units, thus offering a more flexible approach. We discuss the strengths and weaknesses of each approach and illustrate their application using audit data from England and Wales on Adult Cardiac Surgery (ACS) and Percutaneous Coronary Intervention (PCI).

**Results:**

In general, the overdispersion-corrected common-mean model and the random effects approaches produced similar *p*-values for the detection of outliers. For the ACS dataset (41 hospitals) three outliers were identified in total but only one was identified by all methods above. For the PCI dataset (88 hospitals), seven outliers were identified in total but only two were identified by all methods. The common-mean model uncorrected for overdispersion produced several more outliers. The reason for observing similar *p*-values for all three approaches could be attributed to the fact that the between-hospital variance was relatively small in both datasets, resulting only in a mild violation of the common-mean assumption; in this situation, the overdispersion correction worked well.

**Conclusion:**

If the common-mean assumption is likely to hold, all three methods are appropriate to use for outlier detection and their results should be similar. Random effect methods may be the preferred approach when the common-mean assumption is likely to be violated.

**Supplementary Information:**

The online version contains supplementary material available at 10.1186/s12913-022-08995-z.

## Background

The detection and management of outliers when monitoring institutional performance is important in maintaining and improving the quality of health care. In the UK, NHS England monitors the performance of hospitals and individual clinicians to help them identify necessary improvements for patient care. For example, national audit programs exist in Diabetes, Dementia, Lung cancer, Cardiovascular Outcomes, and other fields. Nowadays, outlier detection is an essential aspect of such audits. Patients seek to receive the best possible health care, and government bodies and healthcare providers seek to identify high and low performers to guide quality improvement in clinical care.

The implications of being classified as an outlier can be huge. Low-performing hospitals are likely to face intense scrutiny and patients might choose to avoid low-performing hospitals or surgeons. However, failing to identify outliers with poor performance may jeopardise patient safety.

Outlier methodology may be applied to both measures of processes of clinical care (e.g., waiting times) as well as outcomes of care (e.g., complication rates, procedural mortality). For the purposes of this paper, we use the term ‘unit’ to refer to the entities whose performance is monitored; units can be hospitals, individual hospital clinicians, general practices or general practitioners.

The aim is to identify units whose performance diverges substantially from the expected performance of a group of units or from an externally set target. These units are often said to be ‘outliers’. Depending on the degree of divergence from the performance target, units have been described [1, 2] as ‘normal’, ‘high/low alerts’ or ‘high/low alarms’, with each term describing progressively greater deviation from the performance target. For example, we may want to monitor hospital performance with respect to mortality following cardiac surgery. In this case, the units are the hospitals and each observation within a hospital corresponds to a surgical procedure on an individual patient.

Differences in the performance between units will in part be due to differences in the characteristics of patients in each unit (the unit’s case-mix). For example, when hospitals are compared with respect to in-hospital mortality following a cardiac procedure, it is likely that different hospitals treat patients with different risk profiles. Hospitals treating higher-risk patients would be expected to also have higher proportions of in-hospital deaths (raw mortality). Adjusting for the predicted risk of in-hospital death for each patient can account for some of these differences and help to understand differences in outcomes that are due to quality of care provided. Risk-adjustment is often applied by obtaining the predicted risk for individual patients using a risk model. For example, the predicted risk of in-hospital mortality following cardiac surgery can be obtained using the EuroSCORE risk model [3, 4].

Detecting outliers is an important process with potentially significant implications. Therefore, the ability to detect outliers reliably using appropriate methodology is vital. The principle underpinning all approaches to outlier detection is that a distribution is assumed for the unit-level performance to establish allowable variation in unit performance. Deviations from this distribution indicate outliers. Firstly, the most commonly used approach, the ‘Common-mean model’ [1], uses aggregated unit-level data and is visualised using a funnel plot. It assumes a common true performance for all units, subject to sampling variation. Levels of acceptable variation, the control limits, are constructed around the overall average or an externally set target. However, as explained later, the variability in the data is often higher than that expected under the assumed model, e.g., because of imperfect risk-adjustment or problems with data quality. This is called overdispersion and it may be accounted for by applying a post-hoc correction [5] to the levels of acceptable variation. More recently, the use of random effects models has been proposed to account for the clustered nature of the data within units and overdispersion. A random effects model can be applied to either unit- or individual-level data [2, 6]. The second approach we consider, the ‘Normal-Poisson model’, uses random effects for the units and is applied to unit-level aggregate data. The third approach, the ‘Logistic random effects model’ also uses random effects for the units but is applied to individual-level data (or procedure-level data).

In this paper we provide an overview of these approaches for outlier detection. We examine their corresponding assumptions, discuss their strengths and weaknesses, and review methods for visual representation of the results. For the logistic random effects model, we propose a graphical way to present the results. We illustrate the application of the methods using cardiac data, and include software to implement these approaches in R.

## Methods

### The common-mean model for unit-level data

We consider the case where the individual-level outcome is binary (e.g., death = 1/alive = 0). For unit-level aggregated data, the performance indicator is often taken to be either the proportion, *p*_*i*_ (*i* = 1, …, *K* where *K* denotes the number of units) or the risk-adjusted proportion $\left({p}_i^{ra}\right)$ of events.

The observed proportion of events (${\hat{p}}_i)$ in unit *i*, is the observed number of events (e.g., deaths), *O*_*i*_, divided by the total number of observations in that unit, *n*_*i*_. When a risk model for risk-adjustment is available, each patient within a unit is assigned a predicted risk of having the event. The expected number of events in unit *i*, *E*_*i*_, is calculated by summing the predicted risks for all observations in that unit. The observed risk-adjusted proportion of events $\left({\hat{p}}_i^{ra}\right)$ is equal to the ratio of the observed to the expected number of events, multiplied by the overall proportion of events, $\overline{p}=\frac{\sum {O}_i}{\sum {n}_i}$. If no risk-adjustment is used, then ${E}_i={n}_i\overline{p}$ for all units.

Without loss of generality, we let *p*_*i*_ denote the performance indicator in what follows, where this is either the proportion or risk-adjusted proportion of events. The common population proportion, *p*, is often the overall proportion of events in the sample of all units, $\overline{p}$, and the variance of *p*_*i*_ is ${\sigma}_i^2={p}_i\frac{\left(1-{p}_i\right)}{n_i}$, the binomial variance.

The common-mean model assumes that there exists a *single* underlying true performance, *p*, which is *common* for all units, and that the observed value occurs with variance ${\sigma}_i^2=\frac{p\left(1-p\ \right)}{n_i}$. Using a Normal approximation

1 $${p}_i\sim N\left(p,\frac{p\left(1-p\right)}{n_i}\right),i=1,\dots, K.$$

Any difference between the observed performance in each unit, ${\hat{p}}_i$, and *p* is assumed to be due to random sampling variation. To detect outliers, we test the null hypothesis that the underlying true performance of unit *i*, *p*_*i*_, is equal to the population proportion, *p*:

$${H}_0:{p}_i=p,\kern0.75em \textrm{against}\ \textrm{the}\ \textrm{alternative}$$

2 $${H}_1:{p}_i>p\kern0.5em \textrm{or}\kern0.5em {H}_1:{p}_i<p$$

This can be tested using the following test-statistic:

3 $${Z}_i^{(1)}=\frac{{\hat{p}}_i-p}{\sigma_i}=\frac{{\hat{p}}_i-p}{\sqrt{\frac{p\left(1-p\right)}{n_i}}}.$$

If the null hypothesis is true, ${Z}_i^{(1)}\sim N\left(0,1\right)$. The associated *p*-value for each unit is

4 $${p_{val}}_i^{(1)}=1-P\left({Z}_i^{(1)}\le {z}_i\right)=1-\Phi \left({Z}_i^{(1)}\right).$$

Often, the assumption of a common-mean will be untenable, e.g., due to imperfect risk adjustment. So, in fact, the underlying true proportion of events for each unit is bound to deviate to some extent from the population proportion of events, and consequently the variability in the outcome will be higher than just the random variation in (1). This excess variability is called ‘overdispersion’. Failing to account for overdispersion will mean that the assumed variability is smaller than the variability actually present in the data. This will result in identifying too many units as outliers. Overdispersion in the common-mean model can be accounted for by multiplying the variance with - or adding to it - a corrective overdispersion parameter which may be estimated from the data [1, 5]. For example, if using a multiplicative correction, a value >1 for the overdispersion parameter *φ* indicates that there is unaccounted variability in the performance indicator, i.e. overdispersion is present. Then, the test statistic in (3) is corrected by multiplying the variance under the null hypothesis (denominator term of (3)) by the factor *φ*: $\sqrt{\frac{\varphi\;p\left(1-p\right)}{n_i}}$.

### Visualisation using a funnel plot

The result from applying the common-mean model to a dataset is a *p*-value for each of the units obtained using (4). The *p*-value for a given unit reflects the probability of obtaining the unit’s observed performance if it was actually consistent with the population proportion. A statistically significant *p*-value at a given significance level suggests that unit is an outlier. In the literature [1], units have been usually categorised as: outliers at the *α* = 5% significance level (“Alerts/Better than Expected”), outliers at the *α* = 0.2% level (“Alarms/Substantially better than expected”) and as “Normal” if they are neither Alerts/Better than Expected nor Alarms/Substantially better than expected.

A common way of visualising the results of the outlier process from the common-mean model is a ‘funnel plot’ where the observed value of the performance indicator for a given unit is plotted against a measure of its precision, e.g., the sample size.

For the common-mean model (1), the assumed true proportion of events (known as the target) needs to be set first. The target value could be the overall proportion (or risk-adjusted proportion) of events or an externally set value, *p*. On the vertical axis is the observed proportion (or risk-adjusted proportion) of events and on the horizontal axis the sample size. The target value *p*, is first drawn as a horizontal line. Then, under the assumption that the null hypothesis is true, control limits are drawn around this value for a range of sample sizes, *n*. For a given sample size, *n*, the control limits (potentially with adjustment of overdispersion with the parameter *ϕ*) around the target are $p\pm {z}_{1-\alpha /2}\times \frac{\phi\ p\left(1-p\right)}{\sqrt{n}},n=1,2\dots$. These reflect the range of acceptable variation around the common-mean value at the significance level *a* for a unit of size *n*, assuming the null hypothesis is true. For proportions, the width of the 95 and 99.8% control limits decreases with increasing sample size (with rate $1/\sqrt{n} )$ giving rise to the funnel shape of the graph. The observed values of the performance indicators are then plotted against their size for all units. Units whose observed performance lies beyond the control limits are deemed to be inconsistent with the null hypothesis and hence are denoted as outliers.

### The Normal-Poisson random effects model for unit-level data

The random effects approach relaxes the assumption of model (1) that there is a common underlying true performance for all units and that the variation in the observed performance across units is just by chance. Instead, it assumes that because of imperfect case-mix adjustment or other reasons, the underlying true performance *Y*_*i*_ will differ between units:

5 $${Y}_i\sim N\left(\mu, {\sigma}_i^2+{\tau}^2\right),i=1,\dots, K,$$

where ${\sigma}_i^2$ denotes random variation around population mean *μ*. So, the observed performance of each unit is subject to two sources of variation: the random variation ${\sigma}_i^2$ as for the common-mean model, and additionally an acceptable between-unit variance *τ*^2^.

To detect outliers, we test the null hypothesis that the underlying true performance of unit *i*, *Y*_*i*_, is equal to *μ*:

$${H}_0:{Y}_i=\mu, \kern0.75em \textrm{against}\ \textrm{the}\ \textrm{alternative}$$

6 $${H}_1:{Y}_i>\mu \kern0.5em \textrm{or}\kern0.5em {H}_1:{Y}_i<\mu$$

When the individual-level outcome is binary, a simple Normal random effects [2] model has been used where the performance indicator of interest is the log-relative standardised event ratio (or standardised mortality ratio (SMR) if the event is death), ${Y}_i=\log \left(\frac{O_i}{E_i}\right)$; as before, *O*_*i*_ and *E*_*i*_ denote the observed and expected number of events, respectively. As the distribution of the standardised event ratio *O*/*E* tends to be skewed, the log transformation is used to produce a more symmetric distribution; other transformations are also possible including the square-root of *O*/*E* [7]. Assuming that *O*_*i*_ ∼ *Poisson*(*E*_*i*_), the random variation component of $\log \left(\frac{O_i}{E_i}\right)$ can be approximated by ${\sigma}_i^2=\frac{1}{E_i}.$ The acceptable between-unit variance, *τ*^2^, can be estimated from the data; estimation details are provided in Appendix 1. The population mean, $\mu, \textrm{is}\ \textrm{usually}\ \textrm{log}\left(\frac{\textit{O}}{\textit{E}}\right)$, where O and E denote the sum of the observed and expected values across units, respectively. Because of the assumed distributions, this model is called the *Normal-Poisson random effects model* for unit-level data. The Normal-Poisson model is appropriate as long as the implied Normal approximation holds. This may not hold when the number of events (and unit size) is small; in these situations, further approximations may be necessary [8] or the unit may be excluded from the outlier process.

Under the null hypothesis, model (5) is written as

7 $${Y}_i\sim N\left(\mu, \frac{1}{E_i}+{\tau}^2\right),i=1,\dots, K.$$

The test-statistic for testing the null hypothesis is given by:

8 $${Z}_i^{(2)}=\frac{\log \left(\frac{O_i}{E_i}\right)-\mu }{\frac{1}{E_i}+{\hat{\tau}}^2}$$

leading to the *p*-value ${p_{val}}_i^{(2)}=1-\Phi \left({Z}_i^{(2)}\right)$.

### Visualisation using a funnel plot

A funnel-type plot can also be drawn for the Normal-Poisson random-effects model, similar to that used for the common-mean model. However, the quantities on the axes are different because the assumed null model in (7) is different. For a binary outcome such as in-hospital mortality, the performance indicator on the vertical axis is ${Y}_i=\log \left(\frac{O_i}{E_i}\right)$. This is plotted against a measure of its precision, the expected number of events $\left({\sigma}_i^2=\frac{1}{E_i}\right)$. The target value is usually *μ*= log $\left(\frac{O}{E}\right)$, which is drawn as a horizontal line on the graph. This value will be close to zero as the expected number of events will be usually close to the observed number of events (if the risk model is correctly calibrated in an overall sense). The variance of *Y*_*i*_ under the null hypothesis incorporates two sources of variation: ${\tau}^2+{\sigma}_i^2$. These can be estimated from the data as ${\hat{\tau}}^2+\frac{1}{E_i}$, where ${\hat{\tau}}^2$ is an estimate of *τ*^2^. By varying the expected number of events, the control limits around this target are $\mu \pm {z}_{1-\frac{\alpha }{2}}\times \sqrt{{\hat{\tau}}^2+\frac{1}{E}},E=1,2\dots$, reflecting the acceptable variation around the target value, *μ* under the null hypothesis. Units whose observed performance $\log \left(\frac{O_i}{E_i}\right)$ lies beyond the control limits are outliers.

Most often the quantities presented on the vertical axis are the original $\frac{O}{E}$ ratios, instead of the log$\left(\frac{O}{E}\right)$. The control limits are then:$\exp \left(\mu \pm {z}_{1-\frac{\alpha }{2}}\times \sqrt{{\hat{\tau}}^2+\frac{1}{E}}\right).$

### The logistic random effects model for individual-level data

When the outcome is binary, individuals with *Y* = 1 are said to have experienced the event of interest and individuals with *Y* = 0 to have not. All units will contain multiple observations and these observations are said to be clustered within units; for example, patients might be clustered within hospitals. For clustered data, the binary outcome can be modelled using the *logistic random effects model*, an extension of the well-known logistic regression model*.* The simplest form of the logistic random effects model for *π*_*ij*_ = *P*(*Y*_*ij*_ = 1), with random intercept terms is

9 $$\log\ \left(\frac{\pi_{ij}}{1-{\pi}_{ij}}\right)={\beta}_0+{u}_i,$$

where *β*_0_ is a fixed effect, *u*_*i*_ is the random intercept for unit *i*, and *j* is the indicator for the jth member of unit *i.* In this model, *β*_0_ can be viewed as the average log-odds and the *u*_*i*_^′^*s* correspond to unit-specific deviations from *β*_0_. Usually, it is assumed that ${u}_i\sim N\left(0,{\sigma}_u^2\right)$, where ${\sigma}_u^2$ is the variance of the random intercepts. When the data are clustered, observations within the same unit tend to be more similar than those from different units. The intra-cluster correlation coefficient (ICC) is often used to quantify the degree of similarity; this is also known as the degree of clustering. ICC takes values between 0 and 1, and quantifies the proportion of total variation due to the clustering of patients within units. For binary outcomes, ICC can be estimated by $ICC=\frac{\sigma_u^2}{\frac{\pi^2}{3}+{\sigma}_u^2\ }$ [9].

When a risk model is available, risk-adjustment can be readily incorporated by adding the log-odds of the predicted risk, ${\hat{p}}_{ij},$ for each observation, ${\hat{\eta}}_{ij}=\log \left(\frac{{\hat{p}}_{ij}}{1-{\hat{p}}_{ij}}\right),$ as a covariate:

10 $$\log\ \left(\frac{\pi_{ij}}{1-{\pi}_{ij}}\right)={\beta}_0+{u}_i+{\beta}_1\ {\hat{\eta}}_{ij}$$

Estimates of the random effects are often obtained using Empirical Bayes prediction, where the estimation of the unit-specific effects is effectively a weighted average of the population proportion and the unit proportion (on the log-odds scale); this is the approach we follow in this paper. The implication of using Empirical Bayes prediction for the random effects is that the effects for smaller units tend to be ‘shrunk’ towards the overall average [10, 11]. Model (10) can be fitted in standard software (e.g., R using the function glmer in package lme4 or Stata using the function melogit) to estimate the fixed and random effects.

Under the null hypothesis, all units have random effects from the assumed distribution:

11 $${H}_0:{u}_i\sim N\left(0,{\sigma}_u^2\right)$$

Rejecting the null hypothesis at a given significance level suggests that the random effect for unit *i* is unlikely to be consistent with the random effects distribution under the null hypothesis, i.e., the unit is an outlier.

The test-statistic used for testing *H*_0_ is

12 $${Z}_i^{(3)}=\frac{\hat{u_i}}{S{E}^D\left({\hat{u}}_i\right)},$$

where $\hat{u_i}$ denotes the estimated random effect of unit *i* and ${SE}^D\left({\hat{u}}_i\right)$ the diagnostic standard error [6]. This leads to a one-sided *p*-value ${p_{val}}_i^{(3)}=1-\Phi \left({Z}_i^{(3)}\right)$. It is important to highlight that the hypothesis being tested is whether *u*_*i*_ is consistent with the assumed distribution in (11). Crucially, the diagnostic standard error does *not* represent the precision with which ${\hat{u}}_i$ is estimated.

### Visualisation using a two-panel plot

We now describe an approach to present the results from the outlier process based on an individual-level logistic random effects model.

A two-panel plot is used to present key information about the observed and predicted risks in each unit (left panel) and the results of the outlier process based on the logistic random effects model (right panel). An example of a two panel-plot is given in Fig. 5.

#### Left panel

For the left panel, the units and their sizes are presented on the vertical axis. The observed and predicted risks are on the horizontal axis denoted with the following signs:

- Dashed vertical line: the overall proportion of events across all units.
- Square: the proportion of events in each unit (e.g., in-hospital death after cardiac surgery).
- Cross: the average predicted risk per unit. A low predicted mortality relative to overall mortality across all units, i.e., a ‘cross’ positioned to the left of the population average mortality line indicates that the unit deals with lower risk patients compared to the average.

#### Right panel

For the right panel, the units are also on the vertical axis, and the estimated random effects with intervals for outlier detection are on the horizontal axis, giving rise to a forest plot. This plot includes a vertical line at zero, the ‘target value’ for the random effects. The estimated random effect, ${\hat{u}}_i$, for each unit, and its 100 ×(1 − *a*)% ‘interval for outlier detection’, ${\hat{u}}_i\pm {z}_{1-\frac{\alpha }{2}}\times {SE}^D\left({\hat{u}}_i\right)$, is added as a point and a horizontal bar, respectively. Intervals that do not include the target value of 0, suggest that the corresponding units are outliers at the significance level *a*.

It is important to note that the intervals for outlier detection based on diagnostic standard errors do not represent the precision with which the random effect is estimated, but the evidence that the given unit is an outlier. Hence, their width does not necessarily tend to decrease with increasing hospital size. For each unit, the usual 95 and 99.8% intervals for outlier detection are shown with black and grey solid horizontal bars, respectively. These specify whether a hospital is an outlier at the given significance level.

## Results

### Data

We illustrate the application of the methods described earlier and discuss their results using data from two cardiac audit datasets from England and Wales. The first dataset includes adult patients from 41 hospitals who underwent Cardiac Surgery (ACS). The second dataset includes patients who underwent Percutaneous Coronary Intervention (PCI) at 88 hospitals. Both datasets were obtained for procedures performed during the three-year period April 2015–March 2018 (97,173 procedures in total for the ACS data and 262,035 for the PCI data). The outcome of interest for both datasets was early mortality (in-hospital mortality for ACS, and 30-day mortality for PCI). The average mortality was 1.8% for ACS and 2.7% for PCI. The median number of procedures per hospital (interquartile range) for the ACS data was 2361(1064) and for the PCI data was 2535(2887). For each dataset, the aim is to compare hospitals with respect to mortality to identify outlying hospitals.

### Risk-adjustment models

For both datasets, suitable risk-models were available for risk-adjustment. For the ACS data, a re-calibrated EuroSCORE logistic risk model [3, 4] to predict the probability of in-hospital death has been used (details about the risk factors and the model re-calibration are provided in Appendix 1). For the PCI data, the British Cardiovascular Intervention Society (BCIS) logistic regression model [12] was used to obtain the predicted risk of 30-day mortality (details about the risk factors are provided in Appendix 1).

**Fig. 1:**
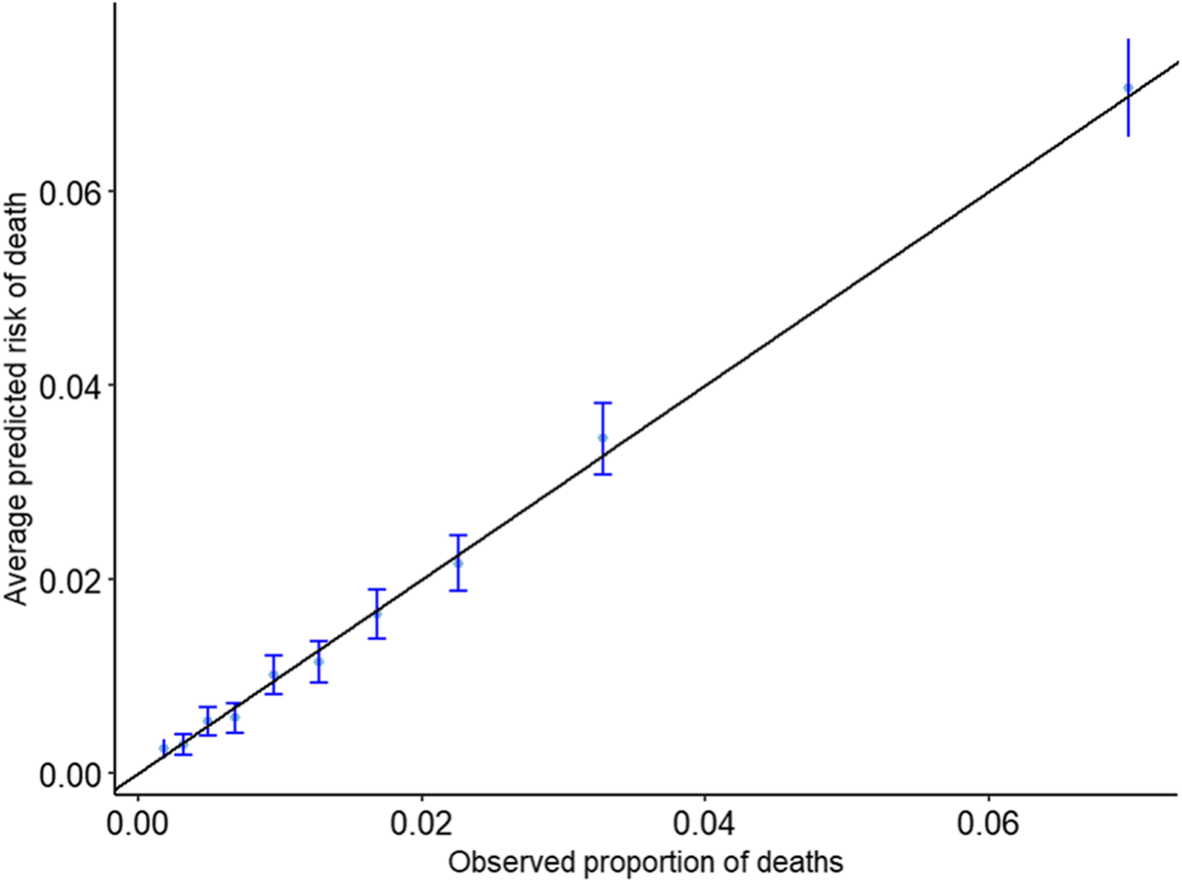
Calibration plot for the ACS data. Observed proportion of deaths against average predicted risk in groups defined by deciles of the predicted risk

We assessed the quality of the models used for risk-adjustment using measures of calibration (calibration slope and calibration in-the-large) and discrimination (C-statistic). A value of 0 for the calibration in-the-large suggests that the average predicted probability is equal to the observed proportion of events. A value of 1 for the calibration slope suggests a perfectly calibrated model. The C-statistic takes values between 0.5 to 1, with higher values meaning higher ability to discriminate between high- and low-risk patients. The estimated model performance measures with 95% confidence intervals are provided in Table 1. The models were well calibrated. This is also confirmed by the calibration plots which show the agreement between the observed proportion of deaths and the average predicted risk in groups defined by deciles of the predicted risks (Fig. 1 and Fig. S1 in Appendix 1 for the ACS and the PCI data, respectively). The model used for the PCI data had a greater discrimination than that used for the ACS data.

**Table 1:** Validation measures for the models used for risk-adjustment.

Risk Model (Dataset)	Calibration Slope (95% CI)	Calibration-in the large (95% CI)	C-statistic (95% CI)
Re-calibrated EuroSCORE (ACS data)	1.00 (0.95, 1.05)	0.00 (− 0.05, 0.05)	0.77 (0.762, 0.784)
BCIS model (PCI data)	1.08 (1.06, 1.09)	0.25 (0.23, 0.28)	0.87 (0.865, 0.874)

### Results: common-mean model

In the plots to follow, unless otherwise stated, it should be assumed that the common-mean model was corrected for overdispersion. Hospitals which are not outliers at either the 5% or 0.02% level are said to be ‘Normal’ (black colour). Outliers at the 5% level are said to be ‘Better than Expected’ (blue) if they perform better than normal and ‘Alerts’(purple) if they perform worse than normal. Outliers at the 0.02% level are said to be ‘Substantially Better than Expected’ (green) if they perform better than normal and ‘Alarms’ (red) if they perform worse than normal.

**Fig. 2:**
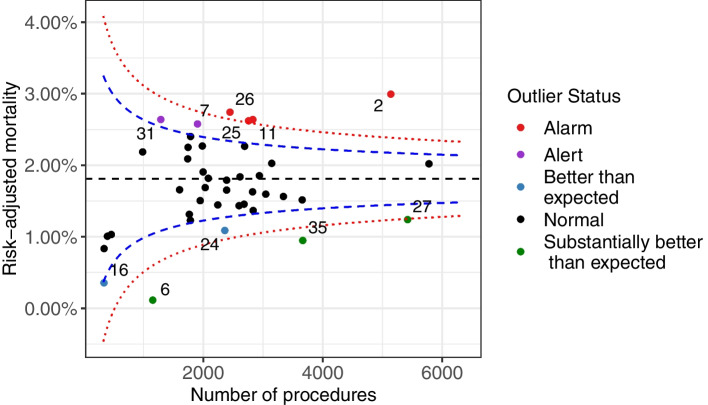
Funnel plot (common-mean model) for the risk-adjusted proportion of events in the ACS data (41 institutions), without correction for overdispersion. Blue and red lines correspond to 95 and 99.8% control limits

**Fig. 3:**
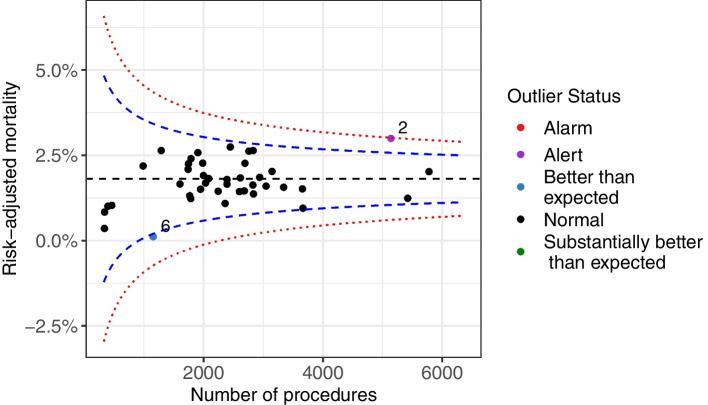
Funnel plot (common-mean model) for the risk-adjusted proportion of events in the ACS data (41 institutions) with correction for overdispersion. Blue and red lines correspond to 95 and 99.8% control limits

Funnel plots based on the common-mean model without and with correction for overdispersion for the ACS data are shown in Fig. 2 and Fig. 3, respectively, where the risk-adjusted proportion of events is on the vertical axis. Figure 2 shows that without correction for overdispersion several units are identified as outliers. Figure 3 shows that after correction for overdispersion there was just one ‘Alert’ (hospital 2) and one hospital ‘Better than expected’ (hospital 6). The overdispersion parameter was estimated to be 4.39 indicating that overdispersion was indeed present. An analogous funnel plot for the PCI data (the overdispersion parameter was 4.45) is presented in Appendix 1 (Fig. S2).

### Results: Normal-Poisson random effects model

**Fig. 4:**
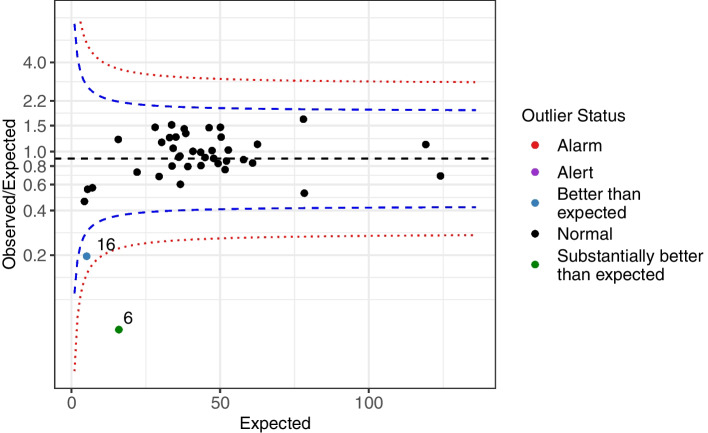
Funnel plot (Normal-Poisson model) for the ratio of observed to expected number of events (O/E) for the ACS data (41 institutions). Presented on the log-scale for the vertical axis. Blue and red lines correspond to 95 and 99.8% control limits

A funnel plot based on the Normal-Poisson random effects model for unit level data for the ACS data is shown in Fig. 4. The estimated between-hospital variance after risk adjustment was $\hat{\tau}=0.38$ ($\hat{\tau}=0.18$ for the PCI data). Figure 4 shows there was one hospital ‘Better than expected’ (hospital 16) and one ‘Substantially better than expected’ (hospital 6). An analogous plot for the PCI data is presented in Appendix 1 (Fig. S3).

### Results: logistic random effects model

The between-hospital variability in the outcome after risk-adjustment was ${\hat{\sigma}}_u=0.28$ and ICC = 0.024 for the ACS data (${\hat{\sigma}}_u=0.20,$ ICC = 0.012 for the PCI data). These figures suggest that the degree of clustering was small in both of the datasets, partially due to the high quality of risk-adjustment.

**Fig. 5:**
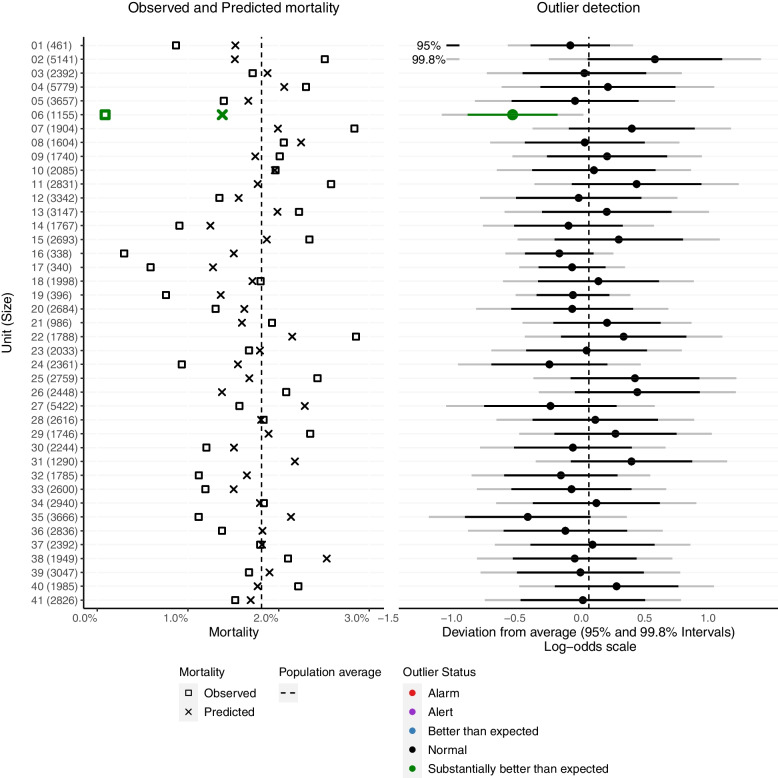
Two-panel plot (logistic random effects model) in the ACS data

The two-panel plot for the ACS data is shown in Fig. 5. It shows that there was just one hospital with a mortality rate ‘Substantially better than expected’ (hospital 6). For this hospital, the observed mortality was markedly lower than the predicted mortality (left panel). An analogous plot for the PCI data is presented in Appendix 1 (Fig. S4) and shows more outlying hospitals.

### Comparison of the results from the three approaches

**Fig. 6:**
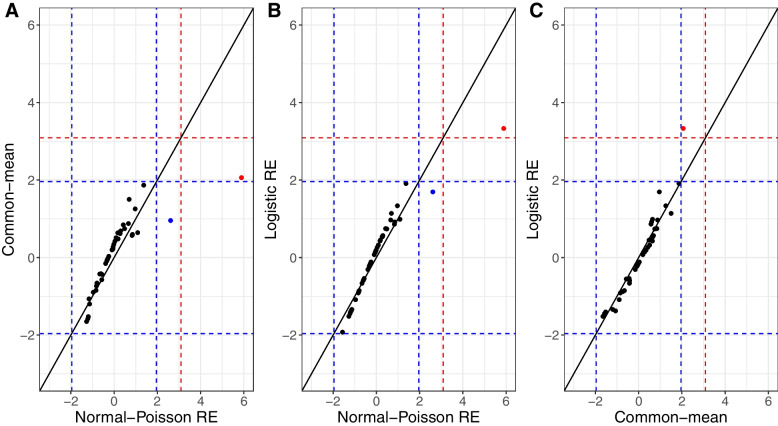
Pairwise comparison of the Z test-statistic values for each method and each hospital in the ACS data

In the analysis of the two cardiac datasets, the results were similar between the two random effects approaches and the common-mean model (Table 2). Figure 6 shows the value of the Z test-statistic for each hospital for each of the pairwise combination of the methods above, showing very high correlation between the Z test-statistic values. This similarity was perhaps to be expected because the between-hospital variance was relatively small in both datasets (ICC < 0.03), resulting in only a mild violation of the common-mean model assumption that the hospitals share a single underlying true performance; consequently, the overdispersion correction for the common-mean model appears to have worked well.

**Table 2:** Results from the outlier process in the PCI and ACS domains using analysis based on hospital or procedure-level data. Each number refers to the ID assigned to an individual hospital.

Data	Outlier Type	Method		
		Hospital-level Common-mean	Hospital-level Normal-Poisson RE	Procedure-level Logistic RE
**ACS (41 units)**	Alarm	–	–	–
	Alert	2	–	–
	Substantially better than expected	–	6	6
	Better than expected	6	16	–
**PCI (88 units)**	Alarm	–	–	–
	Alert	12, 44	44	12, 44
	Substantially better than expected		13, 46	46
	Better than expected	10, 13	36, 58	10, 13, 36, 58

As the variance of the random effects, ${\sigma}_u^2$ (and the between-hospital variance in the Normal-Poisson model, *τ*^2^) increases, one would expect the correlation between the Z test-statistic values from the two random effects approaches to remain very high, and the correlation between the Z test-statistic values from either of the random effects models and the common-mean model to gradually decrease. This hypothesis was confirmed by artificially inducing higher between-hospital variance (*σ*_*u*_ = 0.76, ICC = 0.15) and generating new outcomes for the ACS data. The Z test-statistic values from the two random effect models were very highly correlated between them, and slightly less correlated with the Z test-statistics from the common-mean model (Fig. S5 in Appendix 1).

## Discussion

When comparing the performance of different units with respect to a performance indicator, e.g., risk-adjusted proportion of in-hospital deaths following cardiac surgery, it is often of interest to identify units whose performance deviates from the overall performance across units (outliers). The methods for identifying outliers rely on specifying an underlying assumed model that describes the performance of all units. Any units whose observed performance is found to be inconsistent with the underlying model are denoted outliers.

In this paper we have provided an overview of three of the main methods to identify outliers for binary outcomes: the common-mean model and the Normal-Poisson random effects model for unit-level data and the logistic random effects model for individual-level data. The common-mean model is straightforward to apply, and its results are conveniently visualised via a funnel plot. It also seems to be commonly used in practice. However, it assumes that all units share the same underlying true performance, which may be incorrect, e.g., due to imperfect risk-adjustment. As a result, a correction for overdispersion is important, otherwise it will tend to identify too many outliers. In the two random effects models the common-mean assumption is relaxed, and the units’ underlying true performance is allowed to vary around the common mean. Therefore, the random effects approaches may be more appropriate in most scenarios.

Of the two random effects approaches, the Normal-Poisson random effects model uses aggregated unit-level data, effectively simplifying the data structure. The results can be visualised using a funnel plot. In contrast, the logistic random effects model is applied directly to individual-level data. This will avoid a loss of information if one plans on applying risk-adjustment at the individual level. Therefore, in principle, the logistic random effects model may be considered more appropriate. It is straightforward to implement in standard software and it can readily accommodate risk-adjustment via a risk prediction model, as well as additional individual and unit-level risk factors (e.g., whether a hospital is located in an urban or a rural area) by just including them in the model as explanatory variables.

To identify outliers using the logistic random effects model we followed the approach of Skrondal et al. (2009) [6] for testing based on the diagnostic standard errors. This outlier process may be visualised using the two-panel plot proposed in this paper. Alternative ways of presenting the results from the logistic random effects model also exist. Possibilities include the use of odds ratios or different types of SMRs derived from the logistic random effects model [13]. For example, exponentiating the estimated random effect for a given unit provides the odds of the event for a given patient in that unit over the odds of the event had the patient belonged to the average unit [14].

One issue in the implementation of the random effects approaches is obtaining a value of the variance of the random effects. The variance is often, as it is in this paper, estimated from the data. This, however, may be unduly influenced by a few units with extreme performances, which would ultimately mask their detection as outliers. An alternative approach would be to estimate the variance using a robust estimation procedure that down-weights extreme units, such as Winsorisation or cross-validation [15]. These approaches come with their own challenges, e.g., choosing a suitable proportion for Winsorisation. Another approach would be to set a fixed value for the random effects variance, representing a degree of tolerable variation between units. As an external judgment, it may be specified based on historical data and published before the analysis. Alternatively, expert knowledge may be incorporated into the model via the use of a suitable prior distribution for the between-unit variance (as well as other model parameters) leading to a fully Bayesian approach [16, 17].

In practice, when applying outlier detection methods, there is a chance of a false-positive result or type-1 error. Setting the significance level at a very low value decreases the risk of a type-1 error but also decreases the power to detect a true outlying unit (true positive result). The choice of the significance levels depends on the implications of a false positive result and the importance of identifying true outliers as such.

In our data illustration (cardiac clinical audits) we used two commonly used significance levels for the detection of outliers, 5% (alert) and 0.2% (alarm), which correspond to two different levels for the chance of a false positive result when testing for the outlier status for each unit. The purpose of an alert is to advise that perhaps the standards of care are drifting in the wrong direction. It is not a declaration of an immediate cause for concern, but more a process of flagging up the alert with the hospital/individual concerned. On the other hand, an alarm level means that the result is concerning, and a review process might be activated.

Often the number of units being compared is large. For example, when comparing the performance of cardiac surgeons, the number of units tends to be very large, i.e., several hundred. In this scenario, a large number of surgeons might be identified as outliers due to chance alone. Therefore, it is advisable that a post-processing of the *p*-values be applied to reduce this number. The Bonferroni correction, often used to correct for multiple testing, might be too conservative [18] because reducing the probability of a single false positive test result when the number of units is large, will be at the cost of reducing the power to identify outliers. An alternative strategy is to instead control the False Discovery Rate [19] (FDR) to ensure that the majority of the rejected null hypotheses are correctly rejected.

## Conclusion

Random effect approaches should be the preferred approach when the assumption of the simple common-mean model is unlikely to hold. The logistic random effects model can flexibly accommodate risk-adjustment based on a suitable existing risk model and/or additional risk factors by simply including these factors in the model as explanatory variables. The two-panel plot presented in this paper can be used to visualise the results of the outlier process using the logistic random effects model.

## Supplementary Information

**Additional file 1.**

**Additional file 2.**

## Data Availability

The data that support the findings of this study are available from NICOR but restrictions apply to the availability of these data, which were used under license for the current study, and so are not publicly available. Data are however available from Menelaos Pavlou (m.pavlou@ucl.ac.uk) upon reasonable request and with permission of NICOR.

## Abbreviations

BCIS: British Cardiovascular Intervention Society
ICC: Intra-cluster Correlation Coefficient (ICC)
MLE: Maximum Likelihood Estimation
NICOR: National Institute for Cardiovascular Outcomes Research
PCI: Percutaneous Coronary Intervention
SMR: Standardised Mortality Ratio.
