# Supplementary material for: Outlier identification and monitoring of institutional or clinician performance: an overview of statistical methods and application to national audit data
Source: BMC Health Serv Res. 2023 Jan 10;23:23. doi: 10.1186/s12913-022-08995-z (PMC9832645; doi:10.1186/s12913-022-08995-z)
Supplement: Supplementary file 1 — Additional file 1. [file 12913_2022_8995_MOESM1_ESM.docx]

**Supplementary Material**

by Menelaos Pavlou et al. (m.pavlou@ucl.ac.uk)

**Appendix 1**

# Additional graphs for the PCI data


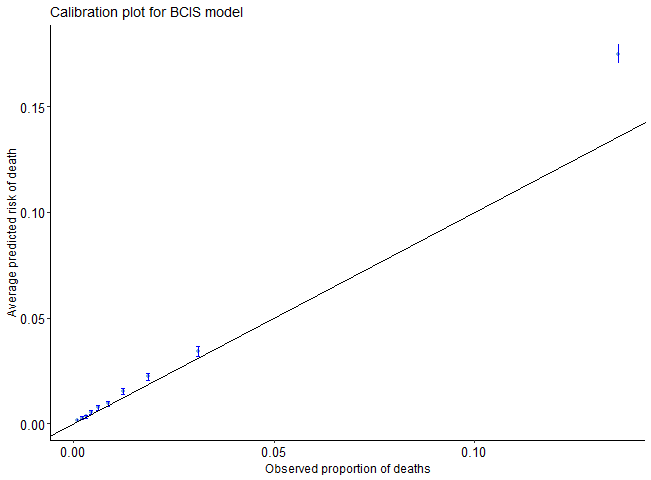


Figure S1: Calibration plot for the PCI data


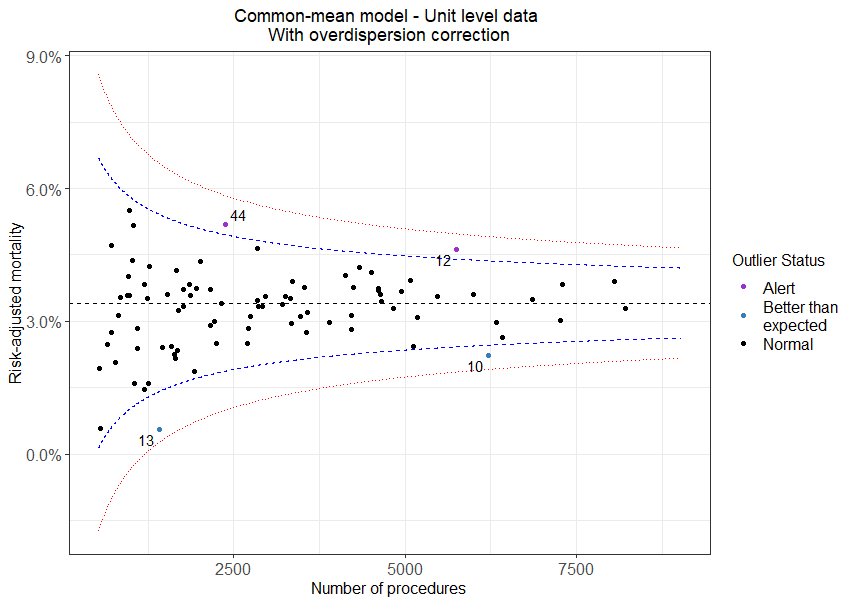


Figure S2. Funnel plot (common-mean model for hospital-level data) for the risk-adjusted proportion of events in the **PCI domain** (88 institutions) with overdispersion correction. Blue and red lines correspond to 95% and 99.8% control limits.


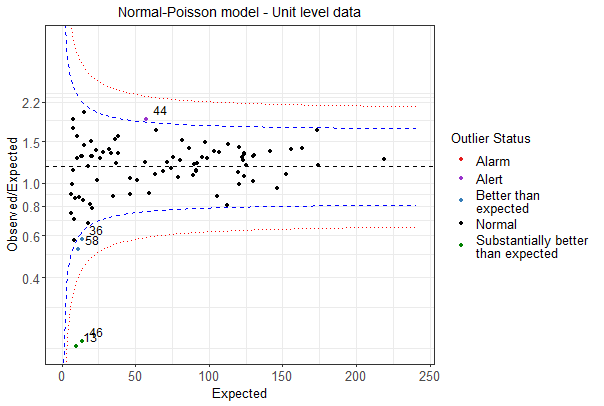


Figure S3. Funnel plot (random effects model for hospital-level data) for the ratio of observed to expected number of events (O/E) in the **PCI domain** (88 institutions). Blue and red lines correspond to 95% and 99.8% control limits.


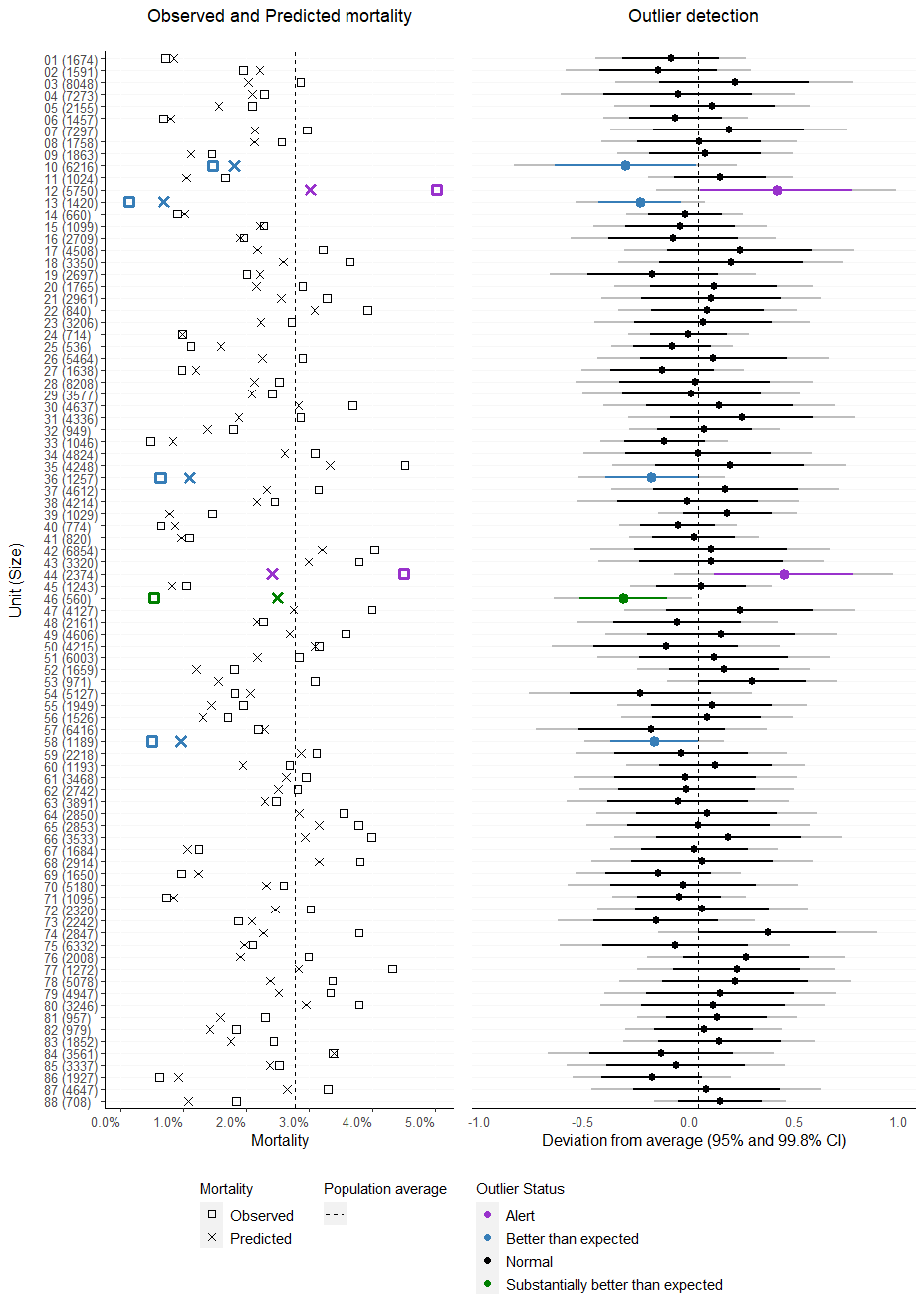


Figure S4. Two-panel plot (random effects model for hospital-level data) in the **PCI data** (88 institutions). Black and grey horizontal bars in the right panel correspond to 95% and 99.8% intervals for outlier detection.


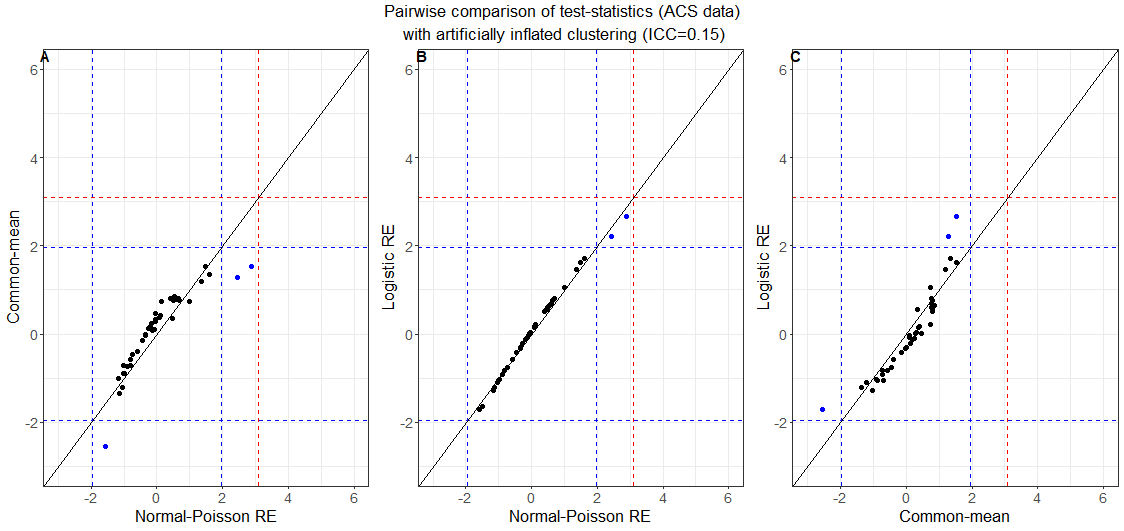


Figure S5. Pairwise comparison of the Z test-statistics for each method and each hospital in the **ACS data** when the degree of clustering was artificially inflated (ICC=0.15)

# 2. Details on the Shrinkage estimator for the random effects model for unit level data

The Der Simonian Laird estimate of $\tau^{2}$ is:

$\hat{\tau}^{2}=\max\left\{ 0,\frac{\sum a_{i}\left( y_{i}-\bar{y}_{\omega} \right)^{2}-\left( m-1 \right)}{\sum a_{i}-\sum a_{i}^{2}/\sum a_{i}} \right\}$.

The ‘shrinkage estimator’ of $\theta_{i}$ is given by:

$\hat{\theta}_{i}=\omega_{i}y_{i}+\left( 1-\omega_{i} \right)\mu$

where $\omega_{i}=\frac{\tau^{2}}{\tau^{2}+\sigma_{i}^{2}}$

with$a_{i}=\frac{1}{\sigma_{\iota}^{2}}\mathrm{and}\bar{y}_{\omega}=\frac{\sum a_{i}y_{i}}{\sum a_{i}}.$

# 3. Comparative versus diagnostic standard errors for the Random effects model for individual-level data

**Empirical Bayes prediction for the random effects**

The posterior distribution, $\omega\left( u_{i} | \boldsymbol{y}_{i}, \boldsymbol{X}_{i};\hat{a},\hat{\beta} \right)$ of $u_{i}$ is obtained by combining two sources of information: the prior distribution of $u_{i}$ , updated with information from the data for the $i^{th}$ cluster, of h the estimates of the fixed effects treated as fixed.

Empirical Bayes prediction is then widely used to assign values,$\hat{u}_{i}$, to the random effects, $u_{i}$ :

$$\begin{aligned} \hat{u}_{i}^{EB}=E\left( u_{i} | \boldsymbol{y}_{i}, \boldsymbol{X}_{\boldsymbol{i}}; \hat{a},\hat{\beta} \right)=\int u_{i} \omega\left( u_{i} | y_{i}, \boldsymbol{X}_{i};\hat{a},\hat{\beta} \right)du_{i}.\#\left( 3 \right) \end{aligned}$$

Two different types of covariance matrices exist for Empirical Bayes predictions of the random effects are useful: the posterior covariance matrices and marginal sampling covariance matrices. The Empirical Bayes standard deviations, called Empirical Bayes standard errors in this context, have two main uses: 1) for inferences regarding the ‘true’ realised values of the random effects for specific institutions (posterior standard deviation) or for model diagnostics (marginal sampling standard deviation).

**Comparative standard errors**

*Posterior standard deviations and prediction error standard deviations* are appropriate for making *inferences about the realised value*$u_{i}$. They can be used for comparing institutions and hence have been given the term *comparative standard errors.*

The *empirical Bayesian posterior variance* of the random effects is:

$$\begin{aligned} \mathrm{var}\left( u_{i} | \boldsymbol{y}_{\boldsymbol{i}}\boldsymbol{,}\boldsymbol{X}_{i};\hat{a},\hat{\beta} \right)=\int\left( u_{i}-\hat{u}_{i}^{EB} \right)\left( u_{i}-\hat{u}_{i}^{EB} \right)^{T}\omega\left( u_{i} | \boldsymbol{y}_{i}, \boldsymbol{X}_{i};\hat{a},\hat{\beta} \right)du_{i}.\#\left( 4 \right) \end{aligned}$$

The prediction error variance is:

$$\mathrm{var}_{\boldsymbol{y}}\left( u_{i}^{EB}-u_{i} | \boldsymbol{X}_{i\boldsymbol{,}}\hat{a},\hat{\beta} \right)=\int\left( u_{i}-\hat{u}_{i}^{EB} \right)\left( u_{i}-\hat{u}_{i}^{EB} \right)^{T}g\left( \boldsymbol{y}_{i} | \boldsymbol{X}_{i};\hat{a},\hat{\beta} \right)du_{i}.$$

It has been shown that

$$\mathrm{var}\left( u_{i} | \boldsymbol{y}_{\boldsymbol{i}}\boldsymbol{,}\boldsymbol{X}_{i};\hat{a} \right)\approx\mathrm{var}_{\boldsymbol{y}}\left( \hat{u}_{i}^{EB}-u_{i} | \boldsymbol{X}_{i\boldsymbol{,}}\hat{a}, \hat{\beta} \right).$$

**Diagnostic standard errors**

The *marginal sampling variance* is defined as:

$$\begin{aligned} \mathrm{var}_{\boldsymbol{y}}\left( u_{i}^{EB} | \boldsymbol{X}_{i\boldsymbol{,}}\hat{a},\hat{\beta} \right)=\mathrm{var}_{\boldsymbol{y}} \left( E\left( u_{i} | \boldsymbol{y}_{i}, \boldsymbol{X}_{i}; \hat{a},\hat{\beta} \right) \right)=var_{\boldsymbol{y}} \int\left( \hat{u}_{i}^{EB} \right)\left( \hat{u}_{i}^{EB} \right)^{T}g\left( \boldsymbol{y}_{\boldsymbol{i}} | \boldsymbol{X}_{i};\hat{a},\hat{\beta} \right)d\boldsymbol{y}_{i} . \#\left( 5 \right) \end{aligned}$$

This can be seen as the variance of the empirical Bayes predictions after repeated sampling of the responses from their marginal distribution, keeping the covariates fixed and using the parameter estimates, $\hat{a}$ and $\hat{\beta}$.

There is no closed-form expression for $\mathrm{var}_{\boldsymbol{y}}\left( u_{i}^{EB} | \boldsymbol{X}_{i\boldsymbol{,}}\hat{a,}\hat{\beta} \right)$ and Skrondal et al. (2009) show that

$$\begin{aligned} \mathrm{var}_{\boldsymbol{y}}\left( u_{i}^{EB} | \boldsymbol{X}_{i\boldsymbol{,}}\hat{a,}\hat{\beta} \right)=\sigma_{u}^{2}-E_{\boldsymbol{y}}\left( \mathrm{var}\left( u_{i} | \boldsymbol{y}_{i}\boldsymbol{,}\boldsymbol{X}_{i};\hat{a}, \hat{\beta} \right) \right), \#\left( 6 \right) \end{aligned}$$

which can be approximated by:

$$\begin{aligned} \mathrm{var}_{\boldsymbol{y}}\left( u_{i}^{EB} | \boldsymbol{X}_{i\boldsymbol{,}}\hat{a},\hat{\beta} \right)\approx\sigma_{u}^{2}- \mathrm{var}\left( u_{i} | \boldsymbol{y}_{i}\boldsymbol{,}\boldsymbol{X}_{i};\hat{a}, \hat{\beta} \right). \#\left( 7 \right) \end{aligned}$$

Skrondal et al. (2009) show via simulation that their proposed approximation works well when the cluster sizes are 20 or more and the intra-cluster correlation coefficient (ICC) is 0.1 or less, or when cluster sizes are more than 100 and the ICC is less than 0.5. For cases where the approximation is unlikely to work well, the marginal sampling variance can be obtained using bootstrapping.

For model diagnostics, such as detecting outlying institutions or checking for violations of the assumption of normality for the random effects, the marginal sampling standard errors can be used and hence they have been termed *diagnostic standard errors*.

**Caveat**

Standard software packages (e.g., glmer function in lme4), by default provide estimates for the comparative standard errors and therefore care should be taken in obtaining the appropriate test-statistic in (8) as the diagnostic and comparative standard errors can be very different[1]. Incorrectly using the comparative standard error for model diagnostics would increase the probability of detecting larger units as outliers. This is because the random effect for large units tend to be estimated with higher precision, and for a large enough unit the confidence interval based on the comparative standard error would be unlikely to include zero. That would simply inform as whether the underlying true random effect is likely to be 0 or not but it would not inform us about whether the true random effect is likely to have emerged from the assumed distribution or not.

# 4. Details on the risk-adjustment models

## 4.1 The re-calibrated logistic EuroSCORE model

According to the EuroSCORE logistic model[2], factors that contribute to the calculation of the predicted risk are age, sex, chronic pulmonary disease, extracardiac arteriopathy, neurological dysfunction, previous cardiac surgery, serum creatinine, active endocarditis, emergency operation, critical preoperative state, ventricular septal rupture unstable angina, LV dysfunction, recent myocardial infarction, pulmonary hypertension, other than isolated coronary surgery, surgery on thoracic aorta.

We here describe the process used within NICOR to obtain a re-calibrated logistic EuroSCORE model for risk-adjustment in the outlier detection process. Since 2011 the predicted risks for patients have been obtained using the re-calibrated model mentioned below and these are the predicted risks used by NICOR for risk-adjustment since then.

Several re-calibration methods were attempted, and the final model was as follows:

logit(π) = a + (1 + b)*logit(ES) + c*logit(ES)2 + d*logit(ES)3 + f

where

• ES = predicted risk using logistic EuroSCORE (a number between 0 and 1)

• logit(π) = log(π) - log(1-π)

- π=Probability of in-hospital death

• a = -1.510967006

• b = -0.4417363

• c = -0.094976301

• d = 0.000509009

- f = a scalar added to the intercept term (essentially recalibration of the intercept term)

such that the overall predicted risk is equal to the proportion of events for that year.

For example,

f = 0 if operation was between 1 April 2011 and 31 March 2012;

f = -0.052220794 if between 1 April 2012 and 31 March 2013;

f = -0.126004173if between 1 April 2013 and 31 March 2014

At the time of re-calibration, the recalibrated model was found to have improved calibration compared to the original model. Hence, this is the risk-adjustment model that has been used by NICOR since then. We used the same model to obtain the predicted risks in this data-illustration.

## 4.1 The BCIS model

Patient characteristics that contribute to the calculation of the predicted risk in the BCIS risk model[3] are: age, sex, diabetes, previous myocardial infarction, renal disease, history of cerebrovascular event, urgency indication and cardiogenic shock. The model also includes interaction terms between urgency indication and cardiogenic shock, age and cardiogenic shock, and age and diabetes.

1. Skrondal, A. and S. Rabe-Hesketh, *Prediction in multilevel generalized linear models.* Journal of the Royal Statistical Society: Series A (Statistics in Society), 2009. **172**(3): p. 659-687.

2. Roques, F., et al., *The logistic EuroSCORE.* Eur Heart J, 2003. **24**(9): p. 881-2.

3. McAllister, K.S., et al., *A contemporary risk model for predicting 30-day mortality following percutaneous coronary intervention in England and Wales.* Int J Cardiol, 2016. **210**: p. 125-32.
